# Death Associated to Methicillin Resistant *Staphylococcus aureus* ST8 Infection in Two Dolphins Maintained Under Human Care, Italy

**DOI:** 10.3389/fimmu.2018.02726

**Published:** 2018-11-22

**Authors:** Sandro Mazzariol, Michela Corrò, Elena Tonon, Barbara Biancani, Cinzia Centelleghe, Claudia Gili

**Affiliations:** ^1^Department of Comparative Biomedicine and Food Science, University of Padua, Padua, Italy; ^2^Department of Diagnostics in Animal Health, Istituto Zooprofilattico Sperimentale delle Venezie, Padova, Italy; ^3^Costa Edutainment spa, Riccione, Italy

**Keywords:** methicillin-resistant *Staphylococcus aureus* (MRSA-ST8), Risso's dolphin, bottlenose dolphin, septicemia, microbiological examinations, (genes codifying) antibiotic resistance

## Abstract

The present study describes the isolation of Methicillin-resistant *Staphylococcus aureus* (MRSA) from respiratory tract of 2 dolphins of different origin, a stranded juvenile Risso's dolphin (*Grampus griseus*) and a captive born common bottlenose dolphin (*Tursiops truncatus*) calf, which died in the same institution at 1-month distance from the other. A complete microbiological and genetic investigation confirmed the presence of MRSA clone-complex 8, sequence type (ST) 8, *spa-*type t008 in both individuals. This strain differs from the one previously reported in walruses and dolphins and has never been described in dolphins before, but it is randomly isolated from Italian human patients. Vertical transmission of the infection may also occurs in other species and considering the description and location of the pathological lesions, this seems to be the most likely route of transmission implied in the young bottlenose dolphin. *Staphylococcus aureus* is known as an opportunistic agent, usually secondary to other pathogens, but its multiple antibiotic resistance and its zoonotic implications suggest a thorough and strict application of animal management hygiene protocols.

## Background

The genus *Staphylococcus* consists of a variety of opportunistic pathogens of variable relevance in veterinary medicine, among which the most clinically relevant staphylococci are the coagulase positive *Staphylococcus aureus*. A noted property of staphylococci is their ability to become resistant to methicillin due to the presence of the *mecA* and *mecC* genes by encoding an altered penicillin-binding protein with low affinity for all beta-lactam antimicrobials (1). Methicillin-resistant *S. aureus* (MRSA) is a significant pathogen that has been identified in the community (community-associated MRSA, CA-MRSA), in hospitals (hospital-associated MRSA, HA-MRSA), and in livestock (livestock-associated MRSA, LA-MRSA), particularly in pigs, and is among the most frequent community and nosocomial-infections in the world (2). From an epidemiological point of view, the relative prevalence of MRSA lineages and their subtypes appear to vary according to the geographical area of detection (2). MRSA is a significant pathogen responsible for a wide range of pathological conditions in animals, including mastitis in cattle, exudative dermatitis in swine, and mild skin infections to severe invasive disease in humans; some diseases related to MRSA infection were also reported in wildlife: more in details, fatal septicemia and dermatitis was reported in Sweden European hedgehogs (3). In this regards, Monecke et al. (4) reported a wide variety of MRSA lineages in German, Austrian and Swedish wildlife even if resistance rates in these strains were rather low. Nevertheless, it has rarely been described in marine mammals (4–7). MRSA has been occasionally reported in free-ranging Atlantic bottlenose dolphins (*Tursiops truncatus*) (8–10), in gray seal (*Halichoerus grypus*), harbor seal (*Phoca vitulina*), and Southern elephant seal (*Mirounga leonina*) (11) and in short-finned pilot whales (*Globicephala macrorhynchus*) (12), although it was also isolated at the necropsy of a bottlenose dolphin maintained under human care (6). In all these cases, MRSA finding was not related to any clinical or pathological change.

## Case presentation

In 2005 a young female Risso's dolphin (*Grampus griseus*) was found stranded in the Ancona harbor (Italy, Central Adriatic) with an adult female, likely the mother. Due to the poor clinical condition of the adult, both animals were recovered in a temporary facility for rehabilitation: entrance examination revealed both animals were positive to *S. aureus* resistant to Amoxicillin and clavulanic acid, but otherwise the calf was clinically healthy, whilst the mother deceased soon after the arrival in the rescue center. After few months in the rehabilitation center the calf was considered unreleasable and moved to a permanent institution where it was subsequently introduced to the 5 common bottlenose dolphins (*T. truncatus*) resident group. Housing condition has been already reported by Gili et al. (7). During its permanence in the pool the animal on different occasions showed clinical signs, including leukocytosis, gastric discomfort, persistent regurgitation, bilateral blepharospasm, and photophobia, that required pharmacological intervention several times along the years with antibiotic and anti-inflammatory drugs. In December 2011 the animal was again showing lack of appetite with mild lethargy and strong regurgitation. Blood analysis showed moderate neutrophilic leukocytosis with hyposideremia (18.600 WBC with 75% neutrophilia- no bands observed, seric iron 49.9 mcg/dl). In January 2012 a progressive body weight loss was noticed along with a catarrhal discharge from the blowhole. Furthermore, in February ultrasound examination showed inflammatory foci located on the left lung for which bronchoscopy was suggested and the animal showed cutaneous raised and hard lesions that became chronic. At the same time, an increase in leukocytes and neutrophils counting compared to reference values for common bottlenose dolphins and Risso's dolphins (13–15) and to the previous data from the same animal was noticed (>25,000 white blood cells with an 83% of neutrophils, 1–3% bands), supporting the hypothesis of an ongoing chronic bronchopneumonia and worsening of general condition with strong electrolytes imbalance (Na > 180 meq/L and Cl > 140 meq/L). *Staphylococcus aureus* was intermittently isolated from the blow along with other bacteria as *E. coli* and *Pseudomonas* spp., *Sphyngomonas* spp., and *Shewanella* spp. Serological examination for dolphin morbillivirus resulted to be negative. X-ray analyses showed inflammatory foci located on the left caudal pulmonary lobe, supporting bronchopneumonia hypothesis. Unfortunately, the situations got worse despite several antibiotic treatments and different therapeutic approaches: a relevant progressive increase in neutrophil leukocytes counting was observed in the last 10 days (the day before death the WBC count was up to 54,000 WBC−89% neutrophils, seric iron 6 mcg/dl) suggesting an ongoing septicemia and the animal died, 7 years after the stranding event. Thirty-two days later (June 2012) also a 9 days old calf common bottlenose dolphin born in the same pool suddenly died showing shortness of apnea the day before death.

A detailed necropsy was carried out on both these two individuals within 24 h from their death and samples for virological, microbiological, and microscopic examination were collected.

**Figure 1:**
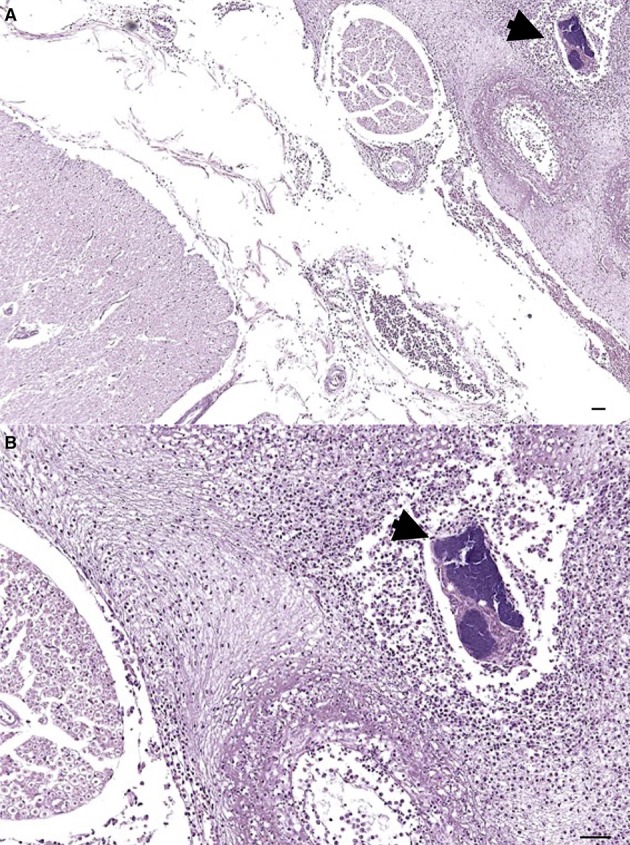
Severe multifocal purulent meningitis in a Risso's dolphin's (*Grampus griseus*) brainstem. On the right, a severe purulent inflammation can be noticed along with a necrotic vessels' wall and a bacterial aggregate (arrowhead). Hematoxylin and Eosin, magnification 4x **(A)** and 10x **(B)**.

The most relevant postmortem findings in Risso's dolphin were a severe multifocal purulent bronchopneumonia developed in the left lung and associated to a foreign body (a decapod's arm accidentally aspired) as well as a chronic inflammatory reaction of the bronchial mucosa. Furthermore, disseminated petechial hemorrhages were grossly evident in several organs and serosal surfaces, while microscopic examination revealed the presence of embolic meningitis (Figure 1), hepatic necrosis and acute splenitis associated to coccoid GRAM-positive bacteria aggregates.

Necropsy on the common dolphin revealed several hemorrhagic and edematous findings observed on pleural and peritoneal serosa and on the epicardium which suggested an acute endotoxic shock, along with a megakaryocytes embolism observed in pulmonary vessels (7).

Molecular analyses for cetacean morbillivirus according to (16) (brain, lungs, spleen, and lymph nodes) and influenza virus type A (lungs, intestine, kidneys) were attempted (17) along with cell culture isolation (spleen, lungs, and brain samples) and electron microscopy (lungs, liver, intestine). All analyses resulted to be negative and no pathological changes related to possible viral infection were observed.

For microbiological examinations, sterile test tubes with swab (Modified Amies Medium, Meus s.r.l. Piove di Sacco, Italy) were used to collect samples from lungs, liver, kidney, spleen, uterus, urine, blood, and brain in the Risso's dolphin during necropsy, while in the common bottlenose dolphin stomach, intestinal tracts, urine, blowhole, and brain were sampled. Swabs were transported to the on-site laboratory immediately after collection and inoculated onto a nutritive medium (Blood Agar Base, Biolife, Milano, Italy) supplemented with 5% defibrinated sheep blood (Allevamento Blood, Teramo, Italy) and onto a nutritive broth (Mueller-Hinton broth, Biokar Diagnostics, Alonne, FR), enriched with 6.5% sodium chloride (Sigma-Aldrich s.r.l., Milano, Italy). After 18–24 h incubation at 37 ± 1°C, the broth culture was seeded onto a methicillin-resistant staphylococci selective medium, (CHROMagar® MRSA II, BD BBL™, Heidelberg, Germany) and incubated at 35 ± 1°C for 24–48 h in aerobic conditions.

Staphylococcal colonies were recognized on nutritive medium, according to colony morphology, Gram stain appearance, catalase, and coagulase tube tests. Suspected pink to mauve colonies grown on the selective medium were confirmed as being MRSA with a multiplex-PCR targeting *nuc* and *mec*A genes (18). *S. aureus* DSMZ 11729 was used as positive control.

MRSAs were isolated from renal, splenic, uterine and meningeal tissues of the Risso's dolphin as well as from the blowhole and several tracts of the intestine (proximal, middle, distal, rectum) of the bottlenose dolphin. Genetic typing of MRSA was further performed by: (i) *spa*-typing (19), (ii) Pulsed-Field Gel Electrophoresis (PFGE) (20), and (iii) Multilocus Sequence Typing (MLST) (21). A multiple detection microarray-based system was also performed on isolates (Miniaturized Microarray, Alere^TM^, Alere technologies, Jena, DE) to identify gene-markers of virulence factors and of antibiotic resistance.

These investigations confirmed the presence of Methicillin-resistant *S. aureus* sequence type (ST) 8, *spa-*type t008 in both animals. PFGE analysis showed a 100% identity of all the analyzed strains. In both individuals, *nuc* gene was identified and the strains showed some genes codifying antibiotic resistance: methicillin-resistance gene *mec*A, the penicillase genes (*bla*Z, *bla*I, *bla*R). However, genes for resistance to tetracycline (*tet*K, *tet*L, *tet*M), vancomycin (*van*A, *van*B), trimethoprim (*tmp*), and chloramphenicol (*cat*) were not found. Strains isolated from the Risso's dolphin were positive for *erm*C and *aad*D encoding, respectively macrolides (erythromycin) and aminoglycosides (tobramycin) resistances; phenotypical resistance to erythromycin was shown also through the agar gel diffusion method. Moreover, all isolates carried capsule (*cap*5) and biofilm associated genes (*ic*aA, *ica*C, *ica*D), as well as proteases and haemolysins genes; none of the isolates showed the *lukS-PV* and *lukF-PV* genes encoding Panton-Valentine leukocidin. Finally, microarray analysis identified *Tursiop*s and *Grampus* strains as Clone Complex 8 (CC8) MRSA-IV, Lyon Clone (*sea*-neg. variant)/WA MRSA-88 with an Assignment Score of 95.83 and 94.20%, respectively.

## Discussion

MRSA infection is a major global healthcare issue for human medicine due to its high rates of morbidity and mortality caused by metastatic bacterial spread or complicated infections as infective endocarditis or sepsis (22). In these cases, the risk of death infections is not related to the strain with non-vancomycin-resistant MRSAs more frequently related to septicemia and subsequent death. Immune system impairment (i.e., old age or organ diseases), the environment (i.e., residence in a nursing home) and the severity of bacteremia are associated with increased risk for death (23).

The cases herein reported support the possible pathogenic role of MRSA also for marine mammals, although such an occurrence should be considered as a rare circumstance, in this case favored by the immune-compromised condition of the two dolphins investigated, respectively related to chronic disease (foreign body aspiration in the Risso's dolphin) and young age (in common bottlenose dolphin). Also, the MRSA circulation in the pool and among the animals should be account as a predisposing factor, with 5 dolphins being positive including the calf bottlenose dolphin's mother (7). As a matter of fact, no pathological changes related to MRSA isolation were reported from previous studies on free-ranging cetaceans' populations (4–6, 8–10). Also in terrestrial wildlife and zoological parks MRSA related diseases have been rarely described with septicemia being reported in European hedgehogs (*Erinaceus europaeus*) in Sweden (3) by CC130-MRSA-XI and in a mongoose species in UK related to Livestock-Associated MRSA CC398 and mecC-positive CC130 (24), two different strains compared to that isolated in the present study.

The complete microbiological and genetic investigation confirmed the presence of MRSA sequence type (ST) 8, *spa-*type t008 in both individuals. This strain differs from the CMRSA2 (USA100) *spa*-type t002 one previously reported in walruses and dolphins by Faires and colleagues in 2009, and has never been described in dolphins. However, MRSA ST-8, spa-type t008 is randomly isolated in Italy among human patients, as well as in animals (25).

Clone Complex 8 is a pandemic lineage and numerous MRSA strains are considered as community and human acquired (respectively, CA and HA-MRSA) (26). In some of these studies, clone type CC8 was indeed reported in animal species, but never in marine mammals (27–30). More in detail, among the CC8-MRSA-IV strain we found the Lyon Clone, also known as UK-EMRSA-2, widely widespread all over France and in other European countries as well. In Australia it was detected in human patients but, never reported in any animal species (26). This isolate did not show the capacity to produce the more common enterotoxins as Panton-Valentine Leukocidine (PVL) (26) as well as the *sea* gene encoding for a superantigenic toxin (31).

ST8 (t008) molecular type has been reported worldwide also within the CC8 and, even if it is known as a human MRSA strain, it was isolated in livestock (LA-MRSA), horses and companion animals, in meat retails and in personnel working in strict contact with these categories (32–36). In addition, van Elk et al. (11) reported the capability of adaptation of ST8 type to the marine environment.

As stated above, *S. aureus* strains resistant to several antibiotic drugs were isolated since 2004 in numerous dolphins living in the same environment (7), although no characterization was performed before the death of the 2 individuals herein reported. Despite the possible influence of alternative vectors of transmission, such as personnel or pool water, and considering the history of these two dolphins, vertical transmission was the most likely route of colonization for the common bottlenose dolphin calf (37). In the case of Risso's dolphin, the anomalous foreign body and the related inflammatory findings in its airway accounted for the prolonged and debilitating condition and may have been the possible route of entry for the bacteria usually hosted in the upper respiratory tract of marine mammals (6, 8–10). Besides this hypothesis, it should be also noted that *S. aureus* was isolated from its blowhole during the first day of rehabilitation as well as from its mother, who died a few days after the admission in the rescue center for causes not related to bacterial infections.

These findings further support the need of a continuous monitoring plan and surveillance activity to be implemented in wild animals, as well as on individuals maintained under human care both in open and closed water systems. Previous studies already expressed a concern for the potential pathogenicity of *S. aureus* in free-ranging cetacean's conservation (9, 38). As suggested by Faires et al. (6), in the case herein a decolonization procedure was performed to prevent any concern for staff and animal care workers. In conclusion, the two dolphins included in our investigation died for a MRSA infection; however, considering that predisposing factors impairing their immune system had played a central role, the isolated bacteria should not be considered as the primary cause of death.

## Ethics statement

The dolphins mentioned in this work were maintained in an artificial environment and handled according to the Italian Zoo Directive law (Dlgs 73/2005); all the samples obtained (*in vivo* diagnostic swabs and blood, and post mortem samples) were collected according to the above and within the Italian D.M. 469/2001, which establishes the management objectives and prescriptions to maintain the species *T. truncatus* under human care.

The Risso's dolphin (*Grampus griseus*), that also belongs to the family Delphinidae, has been sharing the same environment of the other individuals and treated by applying the same husbandry and veterinary principles and best practice protocols as for the common bottlenose dolphins.

## Author contributions

SM and CC performed animal necropsy, tissue and microbiological sampling, and contributed to manuscript writing. BB and CG gave contributions in the acquisition of clinical data for the work, ethical approach for *in vivo* routine diagnostic sampling and contributed to manuscript writing. MC and ET performed microbiological analysis and contributed to manuscript writing. All authors reviewed and agreed on the current version of the manuscript.

### Conflict of interest statement

The authors declare that the research was conducted in the absence of any commercial or financial relationships that could be construed as a potential conflict of interest.
